# Supplementary material for: Optimizing mitochondrial maintenance in extended neuronal projections
Source: PLoS Comput Biol. 2021 Jun 9;17(6):e1009073. doi: 10.1371/journal.pcbi.1009073 (PMC8216566; doi:10.1371/journal.pcbi.1009073)
Supplement: S1 File — Supporting information containing sections ‘Generalization to Branched Axons’ and ‘Contribution of Retrograde Fusion Events’ and accompanying figures Figs A, B and C. (PDF) [file pcbi.1009073.s001.pdf]

Supporting Information: Optimizing mitochondrial maintenance in extended neuronal projections

Anamika Agrawal[1], Elena F. Koslover[1*],

1 Department of Physics, University of California San Diego, La Jolla, California, USA

* ekoslover@physics.ucsd.edu

1 Generalization to Branched Axons

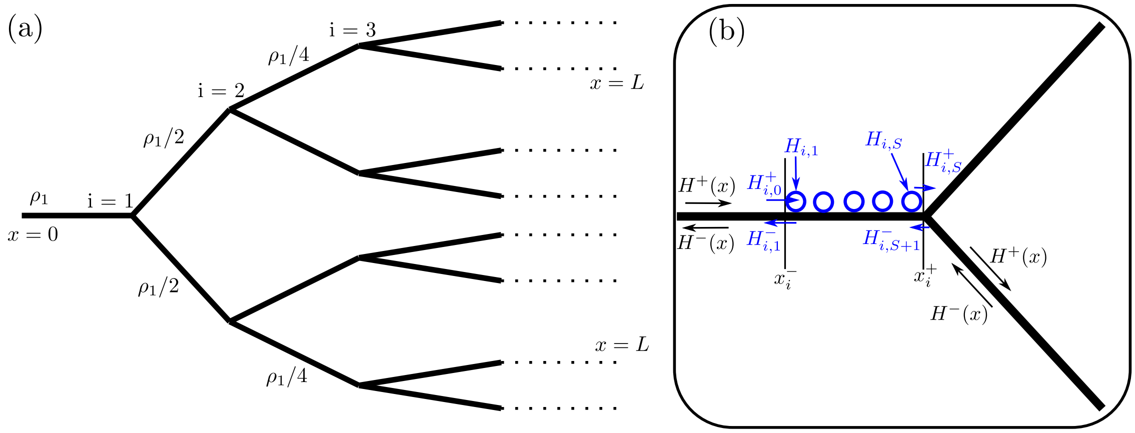

Fig A. (a) Symmetric tree network used for branched axon calculations. Each segment branches into $g = 2$ identical downstream segments. The soma is at position $x = 0$ and distal tips at position $x = L$. (b) Zoomed-in schematic of a demand site at a branching junction. Demand site is located at position x_i, with $S = 5$ stationary mitochondria shown (blue). The health of the jth mitochondrion is given by $H_{i,j}$, and the motile health leaving and entering on each side of the region is labeled. In our simplified model, the demand site is assumed to be infinitely narrow, and $x_i^{\pm}$ refers to the positions in the domain immediately after and before the demand site.

Throughout this work we focus on the interplay between mitochondrial transport, interchange, and aging, assuming a linear geometry to minimize the geometric complexity. However, *in vivo* axons have a tree-like branched structure, with mitochondrial localization observed at the branching points [1]. In this section we present a generalization of the 'Space Station' model for a simple symmetric tree structure with stationary mitochondria localized at the branching points.

We assume that each branching junction splits into $g = 2$ identical downstream branches of equal length. The "demand sites" are placed immediately upstream of each branch point, with an equal number of mitochondria (S) situated at each site. The model geometry is sketched in Fig **??**. The symmetry of the system allows us to define a coordinate system $0 \leq x \leq L$, with 0 corresponding to the soma and L to the distal tips. The motile mitochondria health distribution $H_i^{\pm}(x)$ gives the health density at position x in any one of the corresponding branches. As before, we define $H_{i,j}$ to be the health of the jth mitochondrion at demand site (junction) i. The quantity $H^+_{i,j}$ gives the anterograde-moving health density in the infinitesimally small space between mitochondrion j and $j+1$ at site i; similarly, $H^-_{i,j}$ gives the retrograde-moving health

density between mitochondrion $j-1$ and j. With these definitions, the branched system obeys Equations 13 after replacing the general mitochondrial density ρ with a branch-dependent density ρ_i, defined by

$$\rho_1 = \frac{M - nS}{L}, \quad \rho_{i+1} = \rho_i / g. \tag{S1a}$$

Here ρ_1 is the motile mitochondria density in the initial branch arising from the soma, and this density splits evenly at each junction point to give the downstream density ρ_i between junction $i-1$ and i.

In addition to Eq. 13,the boundary conditions that complete the branched system are:

$$H^+(x_i^-) = H_{i,0}^+, \quad H^+(x_i^+) = H_{i,S}^+/g \tag{S2a}$$

$$H^-(x_i^-) = H_{i,1}^-, \quad H^-(x_i^+) = H_{i,S+1}^-/g \tag{S2b}$$

Eq. S2a indicates that the anterograde health density leaving each junction ($H_{i,S}^+$) splits into g equal branches. Similarly, Eq. S2b defines the retrograde density entering the junction ($H_{i,S+1}^-$) as the sum of retrograde densities from g branches.

The branched model with a tree of depth m has a total of $n = 2^m - 1$ demand sites, with each motile mitochondrion passing m of those sites on its way down the axon. When comparing to the linear model, we compare systems with the same total number of mitochondria M servicing the same number of demand sites n, and with the same distance L from soma to distal tip. It should be noted that the average linear density of motile mitochondria is lower in the branched model because the same total number M is spread out over a larger total branch length $[L_{\text{tot}} = (2^{(m+1)} - 1)L/(m+1)]$. The primary model parameters (decay rate $\widehat{k}_d$, fraction of stopped mitochondria f_s, and average number of stopping events for each protein N_s) are defined to be conceptually analogous to the linear model. As before, we have $\widehat{k}_d = k_d L/v$ and $f_s = nS/M$. Because each mitochondrion traverses only one branch at each level of the tree, the number of stopping events is given by $N_s = 2\widehat{p}_s m$.

The steady-state mitochondrial health at the demand sites in a tree of depth 4-level and 8-level tree are plotted in Fig **??**. The overall value of both average and distal mitochondrial health is somewhat decreased, presumably as a result of the lower density of motile mitochondria servicing the more distal branches. Interestingly, increasing the depth of the branching tree (while keeping a constant length L) only slightly lowers mitochondrial health, despite the fact that the distal density of motile mitochondria decreases exponentially. This result further confirms the observation that the primary relevant parameters are fraction of mitochondria stopped (f_s) and number of stopping events N_s rather than the absolute number of demand sites or density of motile mitochondria. Furthermore, we note that the optimal values of f_s and N_s are largely unchanged in the branched system when compared to the linear geometry (Fig. 5). We therefore conclude that our main results, which rely on a linear axonal geometry, are more generally applicable.

A number of questions remain regarding mitochondrial maintenance in a branching geometry. Namely, the potential effect of redistributing stationary mitochondria at different depths along the tree, the consequences of asymmetric tree geometries, the effect of bidirectional motion into multiple branches, and the role of autophagy in tree-like structures, may further elucidate the optimal strategies for mitostasis in realistic axonal geometries. These more in-depth explorations serve as a promising jumping-off point for future expansion of the model described in this manuscript.

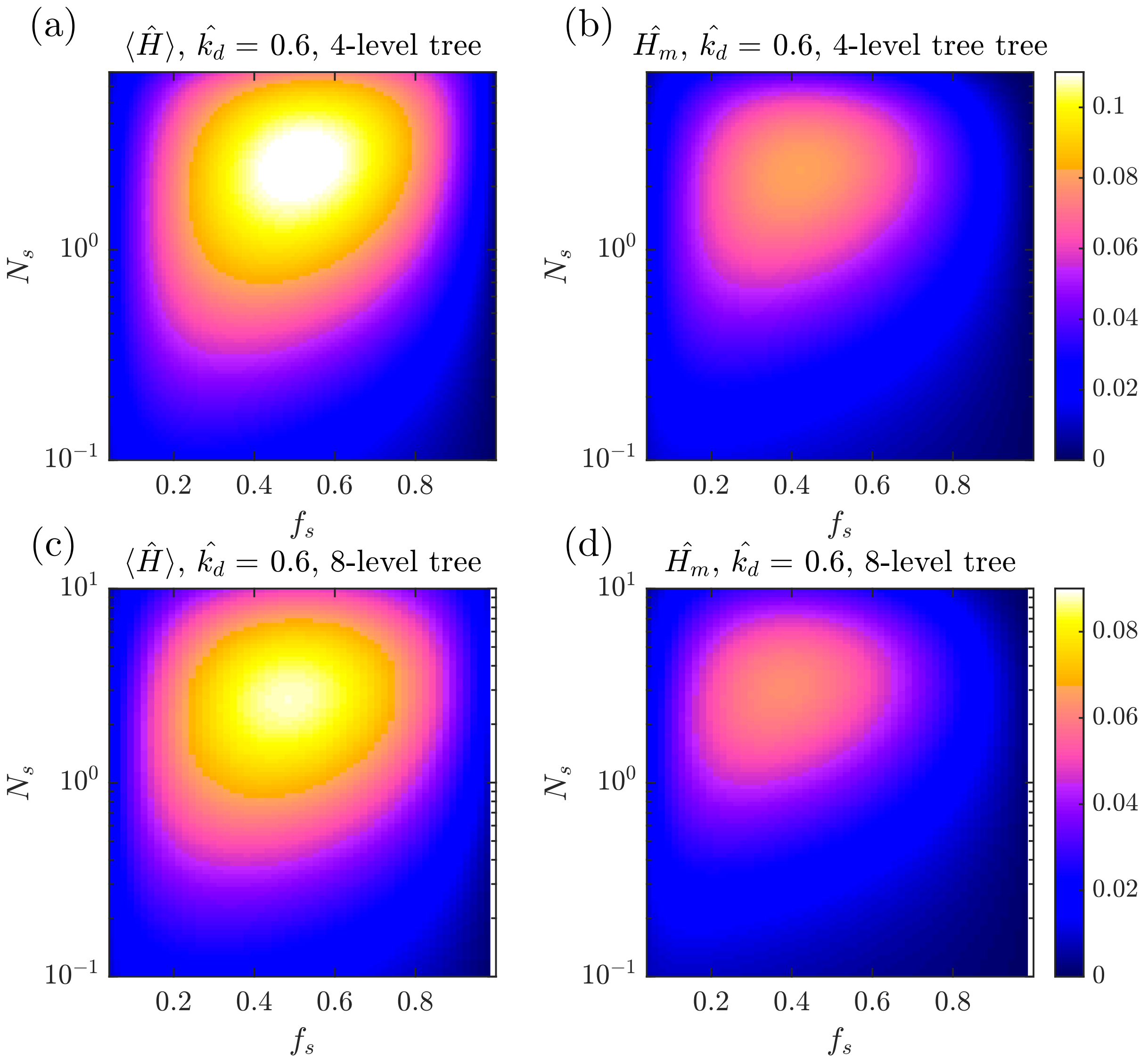

Fig B. Mitochondrial health for expanded model in a symmetric branched tree geometry. (a) Average mitochondrial health as a function of f_s and N_s for a tree of depth $m = 4$, with $n = 15$ demand sites at the branch junctions. (b) Corresponding plot for the health of each distal demand site (furthest from the soma). (c-d) Corresponding plots for a tree of depth $m = 8$, with 255 demand sites.

2 Contribution of retrograde fusion events

Both the SS and CoG models allow health components to transition from a motile to a stationary state in an unbiased fashion. This means mitochonria moving in both the anterograde and retrograde directions are able to stop at demand sites (CoG model) or fuse with stationary mitochondria (SS) model with equivalent stopping or fusion probabilities. Because retrograde-moving mitochondria tend to have lower health levels compared to anterograde-moving mitochondria, removing fusion events between retrograde-moving mitochondria and stationed mitochondria is expected to result in higher health levels at demand sites. Such a modification would be equivalent to mitophagy that is triggered not by mitochondrial health levels but rather by the arrival of mitochondria at the distal terminus.

The 'Space Station' model equations can be modified as follows to reflect a potential regulatory mechanism that completely prohibits fusion of retrograde-moving mitochondria:

$$\frac{dH^{\pm}}{dt} = \mp v \frac{\partial H^{\pm}}{\partial x} - k_d H^{\pm} \tag{S3a}$$

$$\frac{dH_{i,j}}{dt} = \frac{v p_f}{2} \left[H^{+}_{i,j-1}\right] - \left[\frac{v \rho p_f}{4} + k_d\right] H_{i,j} \tag{S3b}$$

$$H^{+}_{i,j} = H^{+}_{i,j-1}\left(1 - \frac{p_f}{2}\right) + \frac{\rho p_f}{4} H_{i,j}, \quad H^{-}(x_{i,j}) = H^{-}_{i,j+1} \tag{S3c}$$

$$H^{+}(L) = H^{-}(L) \tag{S3d}$$

$$v H^{+}(0) = k_p. \tag{S3e}$$

In Eq. S3b, the quantity $\rho/2$ gives the density of anterograde-moving mitochondria, replacing ρ in the original 'Space Station' equation (Eq. 13b).

The steady-state mitochondrial health in the absence of retrograde fusion is plotted in Fig **??**. We see that removing fusion with retrograde mitochondria means that there is no longer an optimum in the number of interaction events between motile and stationary mitochondria. The disadvantage to high N_s in the original model arose from the fusion of unhealthy retrograde mitochondria picking up proteins from stationary organelles and carrying them prematurely back to the soma for recycling. This disadvantage is no longer present if retrograde mitochondria are incapable of fusion.

Removal of retrograde fusion also results in a 50% increase in average health at demand sites (Fig **??**a), again by preventing fusion of the less healthy retrograde mitochondria with stationary organelles at the proximal sites. Interestingly, the maximum health at the most distal site remains largely unchanged (Fig **??**b).

These results indicate that any cellular mechanism capable of biasing exchange events between the motile and stationary population so that retrograde mitochondria were less likely to fuse or stop could be beneficial for enhancing mitochondrial health in the domain.

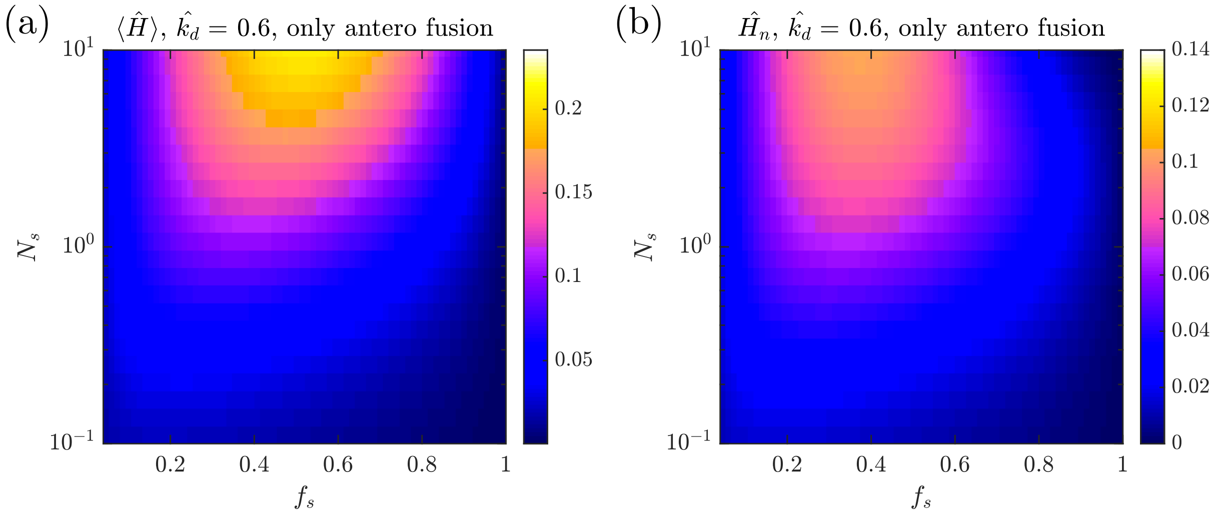

Fig C. Mitochondrial health without retrograde fusion; plots analogous to Fig. 5. (a) Average health across all demand regions as a function of fraction of stopped mitochondria (f_s) and number of stopping events (N_s), for high decay rate ($\widehat{k}_d = 0.6$). (b) Corresponding mitochondrial health at the most distal demand site.

References

1. Misgeld T, Schwarz TL. Mitostasis in neurons: maintaining mitochondria in an extended cellular architecture. Neuron. 2017;96(3):651–666.
