# Supplementary figures and images for: Optimizing mitochondrial maintenance in extended neuronal projections
Source: PLoS Comput Biol. 2021 Jun 9;17(6):e1009073. doi: 10.1371/journal.pcbi.1009073 (PMC8216566; doi:10.1371/journal.pcbi.1009073)

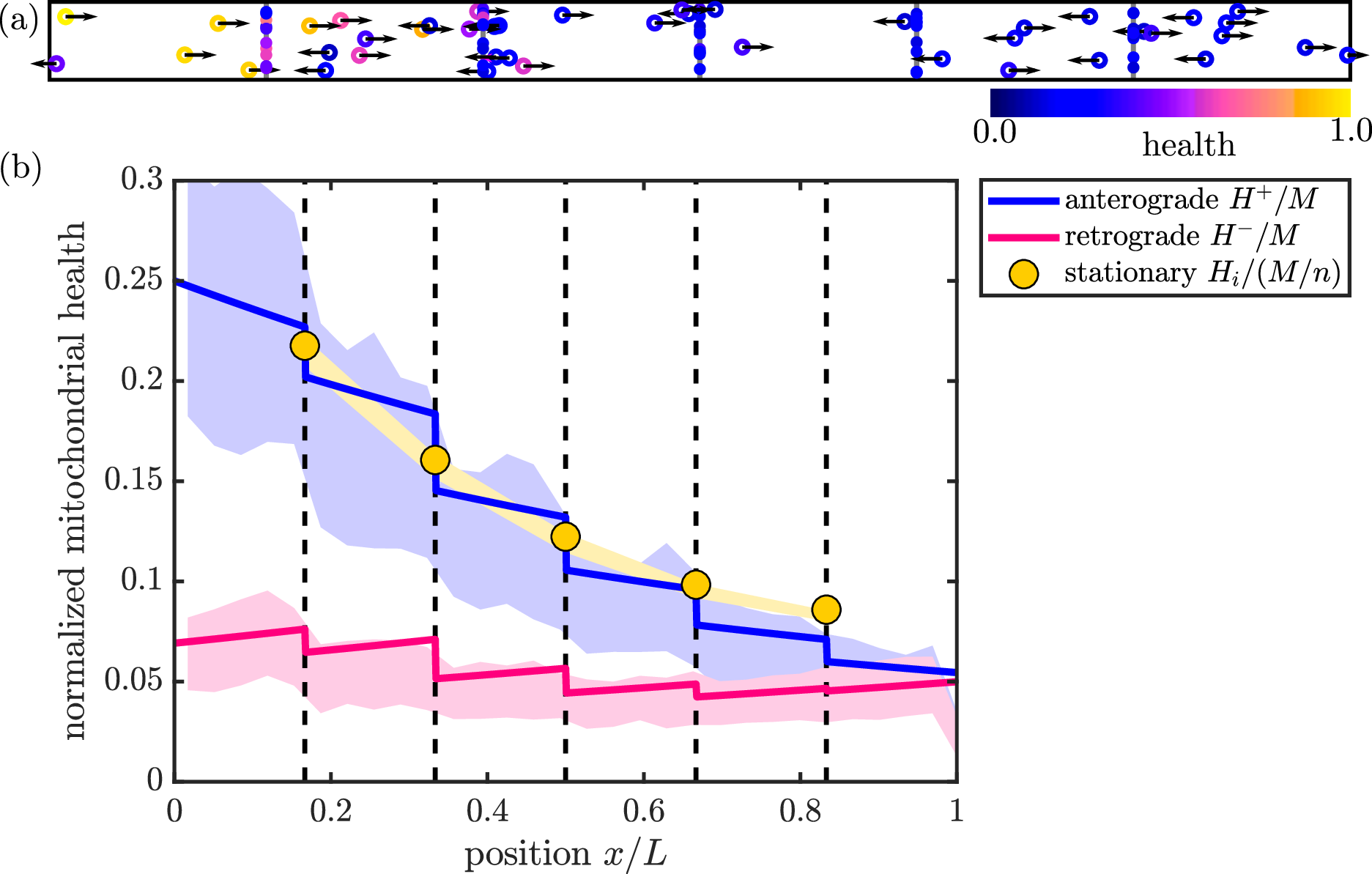

Supplement: S1 Fig — (a) Snapshot of a stochastic simulation for the SS model, with M = 100. (b) Steady-state solution for mitochondrial health in the SS model. Solid curves show linear density of mitochondrial health in anterograde (blue) and retrograde (magenta) mitochondria, normalized by total number of mitochondria in the domain. Yellow circles show total health at each of the discrete demand sites (dashed black lines), normalized by the total number of mitochondria per region. Shaded regions show corresponding quantities from discrete stochastic simulations (mean ± standard deviation) with M = 1500 mitochondria in the domain. Parameters used in (a) and (b): n = 5, ps = 0.4, fs = 0.5, kd L/v = 0.6. (TIF) [file pcbi.1009073.s002.tif]

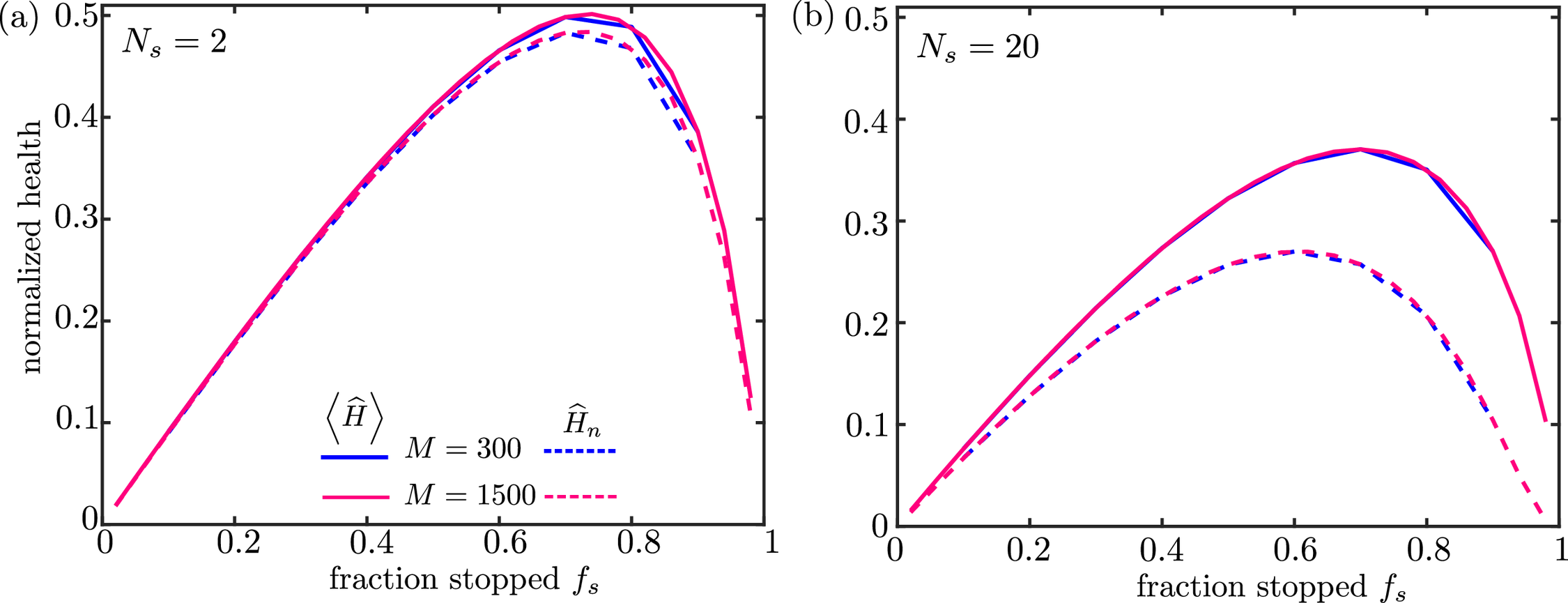

Supplement: S2 Fig — Mitochondrial health as a function of key dimensionless parameters. Solid curves show normalized average health over all demand sites; dashed curves show normalized health at the most distal site. The total number of mitochondria is set to M = 300 (blue) or M = 1500 (magenta). For each fraction of stationary mitochondria the fusion probability is adjusted to give a fixed number of stopping events for an individual protein traversing the domain: (a) Ns = 2 and (b) Ns = 20. All values shown are for the SS model, with n = 30 and k^d=0.06. (TIF) [file pcbi.1009073.s003.tif]

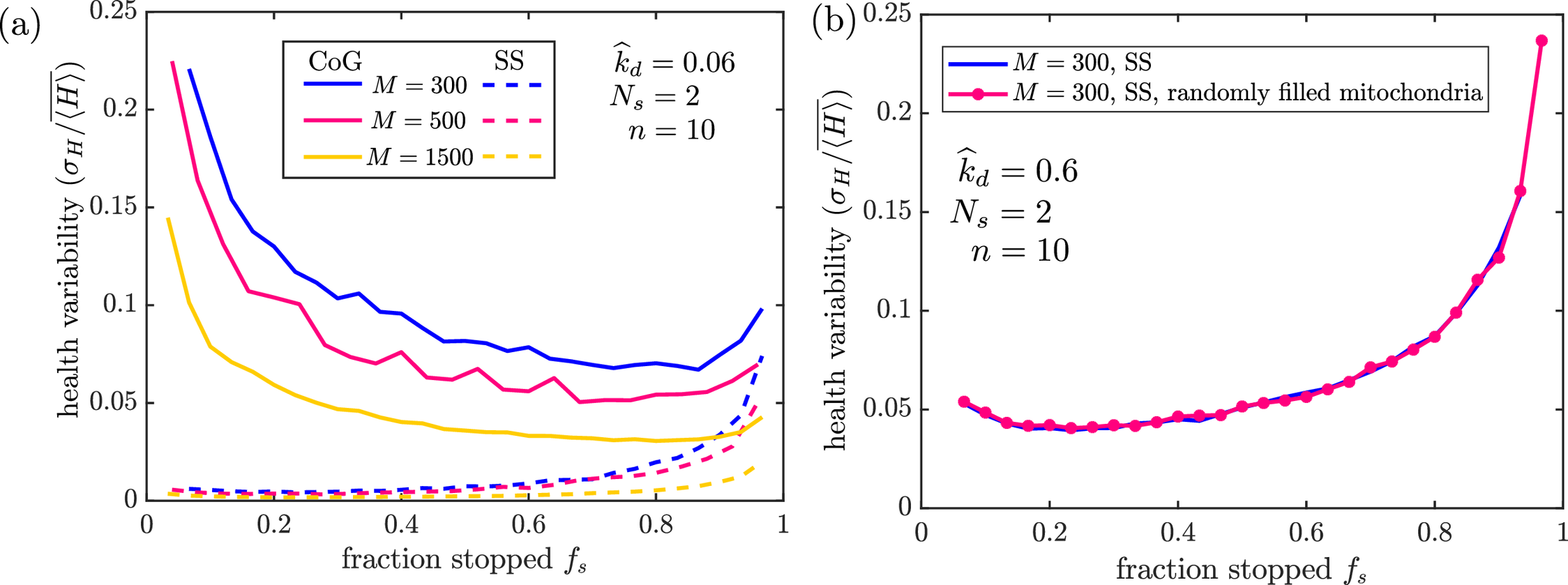

Supplement: S3 Fig — (a) Variability of mitochondrial health in different maintenance models, with low dimensionless decay rate k^d=0.06. Results are shown for 300 (blue), 500 (magenta), and 1500 (yellow) average mitochondria in the domain. Solid lines correspond to simulations of the CoG model and dashed lines to the SS model. (b) Health variability in the SS model does not depend on how stationary mitochondria are distributed among sites. Results plotted are for decay rate k^d=0.6, and number of mitochondria M = 300. Blue: equal number of mitochondria are placed at each demand site. Magenta: position of each stationary mitochondrion is selected uniformly at random among the demand site locations. Each iteration starts from an independently selected mitochondrial distribution. In all plots, variability is computed over 1000 independent iterations of stochastic simulations, using n = 10 demand sites and average stopping number Ns = 2. (TIF) [file pcbi.1009073.s004.tif]

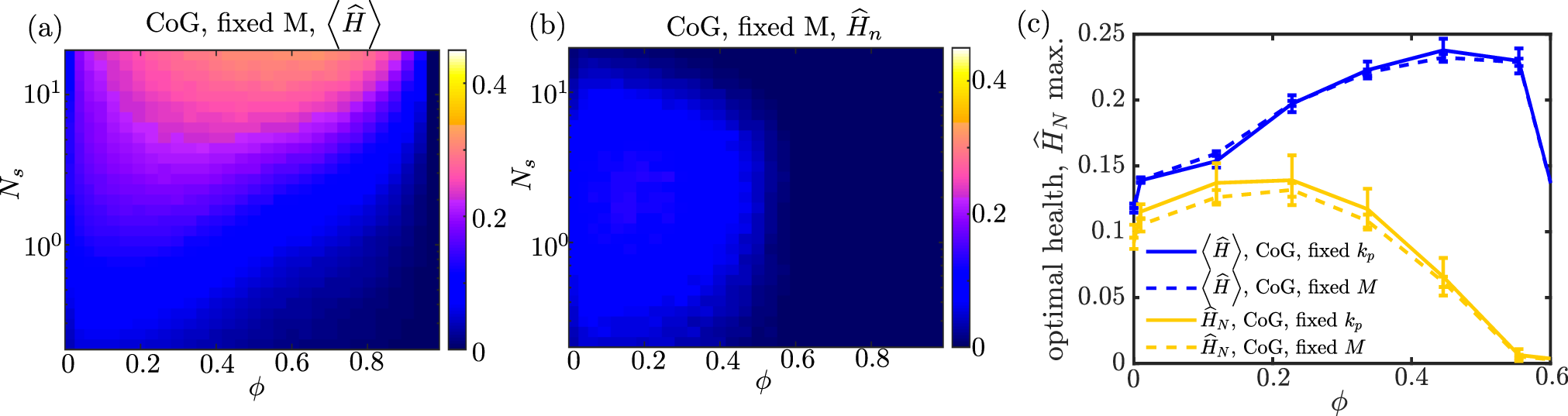

Supplement: S4 Fig — Rescaling health levels by total mitochondrial content is equivalent to a model with explicitly fixed number of mitochondria. (a-b) Average health across all regions (a), and health of last region (b), for simulations of a modified CoG model with fixed M = 150, to be compared to Fig 9b–9c. Health levels are normalized by M/n for both models. (c) Average health (blue) and last region health (yellow) for parameters that optimize the last region health at each fixed value of ϕ. Solid lines show the CoG model with kp values fixed as the mitophagy threshold is varied (identical to curves in Fig 10b). Dashed lines show corresponding plots for the model with explicitly fixed number of mitochondria M. Health levels normalized by M/n are shown to be largely the same in both cases. (TIF) [file pcbi.1009073.s005.tif]

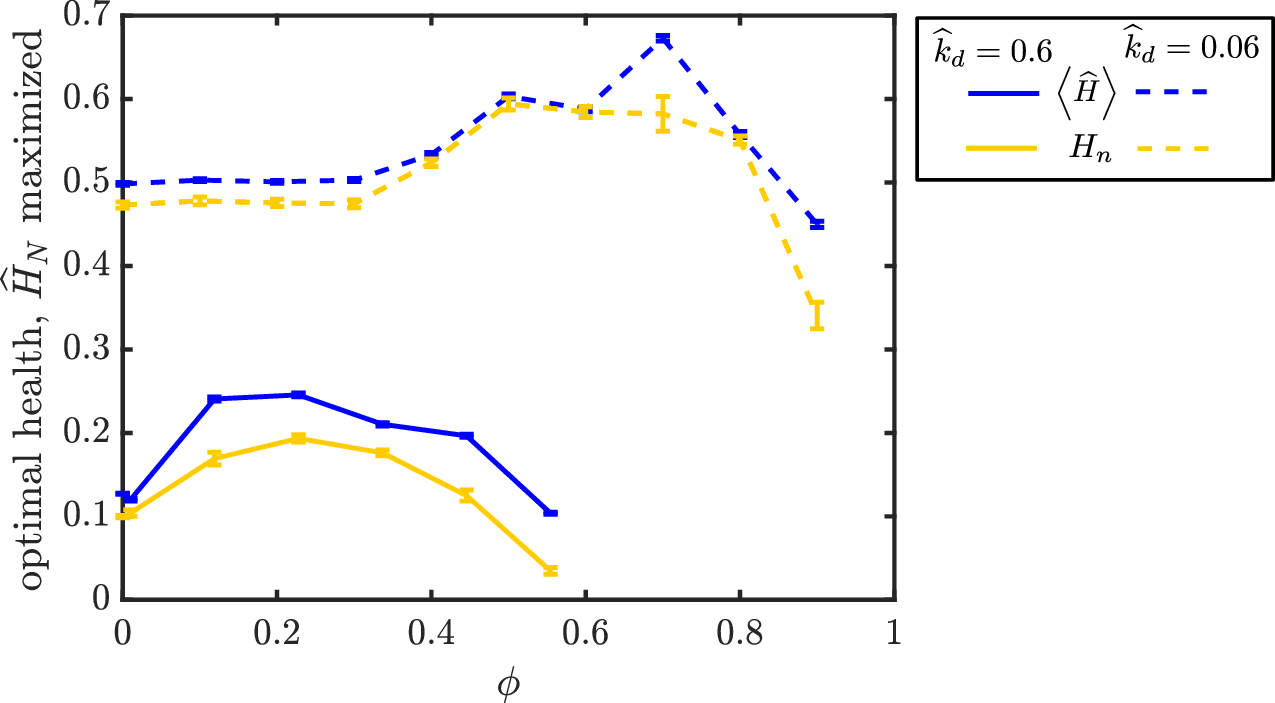

Supplement: S5 Fig — Optimal performance SS model in the presence of mitophagy, for different health decay rates. For each value of ϕ, the parameters fs and Ns were optimized to yield the maximal health in the most distal region. Plotted are the resulting normalized health levels in the distal region (yellow) and averaged over all regions (blue). The health decay rate was set to k^d=0.6 (solid) and k^d=0.06 (dashed). Lower decay rates correspond to a higher mitophagy cutoff value ϕ that yields the maximal overall health. All health levels are normalized by total mitochondrial content per region (M/n), corresponding to a system where the total amount of mitochondrial material is limited. Error bars show standard error of the mean from 10 independent replicates. (TIF) [file pcbi.1009073.s006.tif]

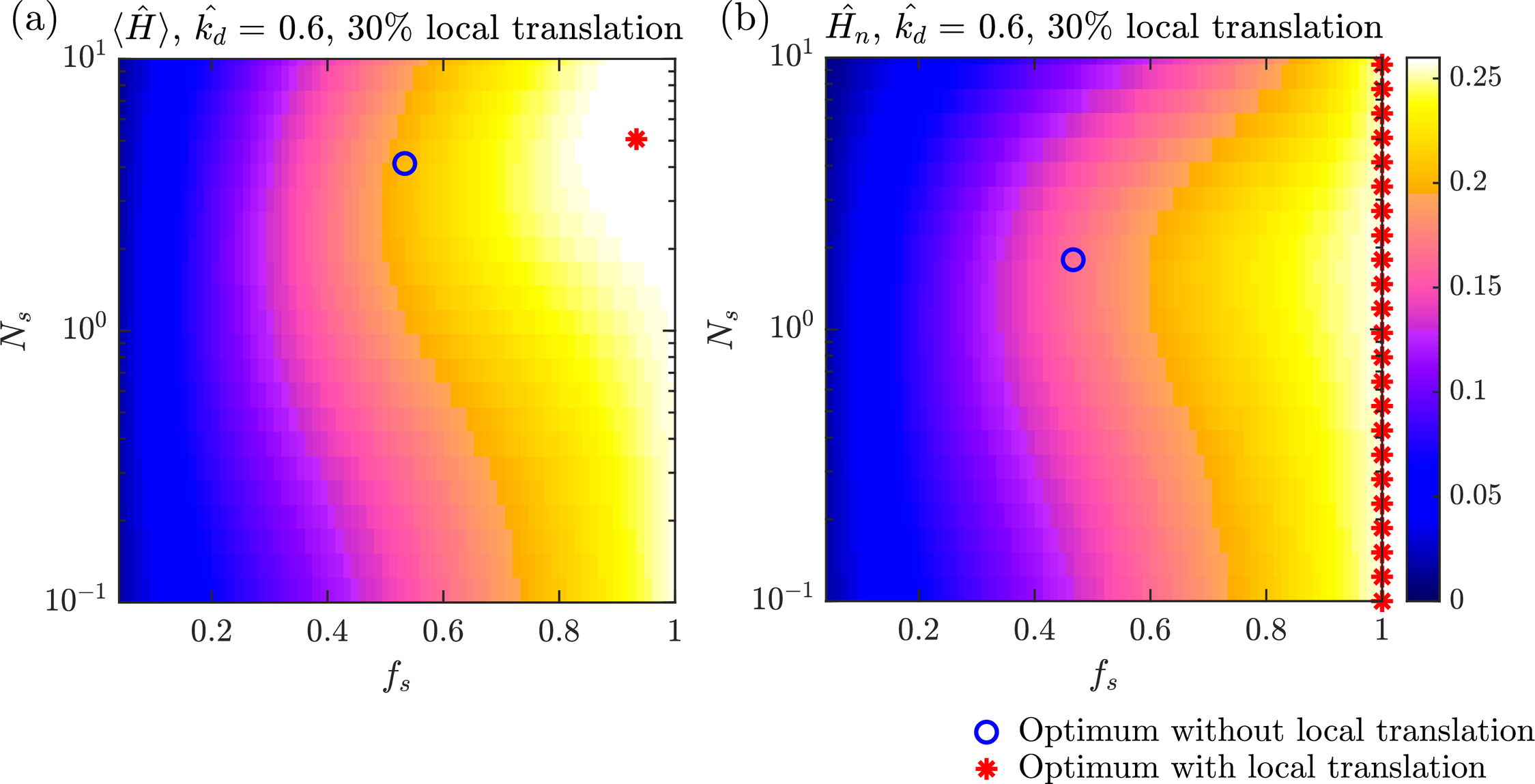

Supplement: S6 Fig — Mitochondrial health in the presence of substantial levels of local translation, with α = 30%. (a) Average health across all demand regions as a function of fraction of stopped mitochondria (fs) and number of stopping events (Ns). Markers show parameters for optimal average health with local translation (red asterisk) and without it (blue open circle). (b) Corresponding plot for normalized mitochondrial health at the most distal demand site. Optimal distal health is obtained by placing all mitochondria in the stationary population (fs = 1), so that health maintenance relies entirely on local translation rather than delivery from the soma. (TIF) [file pcbi.1009073.s007.tif]
